# The relevance of a low *JAK2*^V617F^ allele burden in clinical practice: a monocentric study

**DOI:** 10.18632/oncotarget.16744

**Published:** 2017-03-31

**Authors:** Margherita Perricone, Nicola Polverelli, Giovanni Martinelli, Lucia Catani, Emanuela Ottaviani, Elisa Zuffa, Eugenia Franchini, Arbana Dizdari, Dorian Forte, Elena Sabattini, Michele Cavo, Nicola Vianelli, Francesca Palandri

**Affiliations:** ^1^ Department of Experimental, Diagnostic and Specialty Medicine, Institute of Hematology ‘L. and A. Seràgnoli’, University of Bologna, S. Orsola-Malpighi Hospital, Bologna, Italy; ^2^ Haematopathology Unit, Department of Experimental, Diagnostic and Specialty Medicine, S. Orsola-Malpighi Hospital, University of Bologna, Bologna, Italy

**Keywords:** JAK2, V617F mutation, allele burden, myeloproliferative neoplasms, MPN

## Abstract

Since low *JAK2*^V617F^ allele burden (AB) has been detected also in healthy subjects, its clinical interpretation may be challenging in patients with chronic myeloproliferative neoplasms (MPNs). We tested 1087 subjects for *JAK2*^V617F^ mutation on suspicion of hematological malignancy. Only 497 (45.7%) patients were positive. Here we present clinical and laboratory parameters of a cohort of 35/497 patients with an AB ≤ 3%.

Overall, 22/35 (62.9%) received a WHO-defined diagnosis of MPN and in 14/35 cases (40%) diagnosis was supported by bone marrow (BM) histology (‘’Histology-based’’ diagnosis). In patients that were unable or refused to perform BM evaluation, diagnosis relied on prospective clinical observation (12 cases, 34.3%) and molecular monitoring (6 cases, 17.1%) (‘’Clinical-based’’ or ‘’Molecular-based’’ diagnosis, respectively). In 11/35 (31.4%) patients, a low *JAK2*^V617F^ AB was not conclusive of MPN. The probability to have a final hematological diagnosis (ET/PV/MF) was higher in patients with thrombocytosis than in patients with polyglobulia (73.7% vs 57.1%, respectively). The detection of AB ≥ 0.8% always corresponded to an overt MPN phenotype. The repetition of *JAK2*^V617F^ evaluation over time timely detected the spontaneous expansion (11 cases) or reduction (4 cases) of *JAK2*^V617F^-positive clones and significantly oriented the diagnostic process.

Our study confirms that histology is relevant to discriminate small foci of clonal hematopoiesis with uncertain clinical significance from a full blown disease. Remarkably, our data suggest that a cut-off of AB ≥ 0.8% is very indicative for the presence of a MPN. Monitoring of the AB over time emerged as a convenient and non-invasive method to assess clonal hematopoiesis expansion.

## INTRODUCTION

In 2008, the World Health Organization (WHO) classification indicated the positivity of the *JAK2*^V617F^ mutation as a major criterion for the diagnosis of chronic myeloproliferative neoplasms (MPNs), specifically Essential Thrombocytemia (ET), Polycythemia Vera (PV) and Myelofibrosis (MF) [1–7]. The *JAK2*^V617F^ mutation is detected in around 50–60% of ET and MF patients and in most (95%) patients with PV [8, 9]. Also, the *JAK2*^V617F^ mutation may be found in other hematological malignancies. Infrequent occurrence of this unique *JAK2* mutation has been reported in chronic myelomonocytic leukemia (CMML), atypical or unclassified myeloproliferative disorder (MPD), myelodysplastic syndrome (MDS), systemic mastocytosis (SM), and chronic neutrophilic leukemia (CNL) [10–16]. A *JAK2*^V617F^ allele burden (AB) above 50% identifies patients with a higher thrombotic risk, both in ET and in PV [17–22]. Conversely, a low AB seems to correlate with significantly shortened survival and leukemia-free survival in MF [23–25]. As a result, the determination of the mutation load is becoming a standard diagnostic procedure in most molecular laboratories, though WHO criteria do not specify a cut-off value for the diagnosis of a MPN. In the last years, the extensive and generalized use of molecular techniques with high sensitivity, specifically allele-specific Real-Time Quantitative Polymerase Chain Reaction (RQ-PCR), has significantly increased our ability to detect small mutated clones, with AB below 1% [26–32]. Many recent studies have shown that a small clonal hematopoiesis may be present also in otherwise healthy subjects at low level (0.03–1%) [33–38]. Accordingly, a study of the Myeloproliferative Neoplasms and Related Disorders European Network (MPN&MPNr-EuroNet) on 36 subjects carrying low *JAK2*^V617F^ AB has further suggested that the detection of a small *JAK2*^V617F^-mutated clone cannot represent a sufficient evidence to establish malignant myeloproliferation [39]. As a result, the clinical interpretation of a low AB of the *JAK2*^V617F^ mutation may be challenging. Here, we analyzed the results of *JAK2*^V617F^ molecular tests performed at our Hematology Department in subjects with a suspected hematological disease over a 2-year period. Specifically, we analyzed clinical and laboratory data of patients with a suspected MPN that presented a low (≤ 3%) *JAK2*^V617F^ mutation burden, with the aim to define the frequency and the significance of a low AB in the everyday clinical management.

## RESULTS

### Study plan

We tested 1087 subjects for *JAK2*^V617F^ mutation due to clinical suspicion of hematological malignancy. A total of 716 (65.9%) out of 1087 tests were performed due to a suspect of classical MPN, including ET (299 cases, 41.8%), PV (272 cases, 38%), MF (133 cases, 18.6%) and MPN underling atypical splanchnic vein thrombosis (12 cases, 1.6%). The remaining 371 (34.1%) out of 1087 tests were performed on suspicion of Myeloproliferative Disease (MPDs; 23 cases) or other causes/not specified (348 cases) (data not shown).

**Figure 1:**
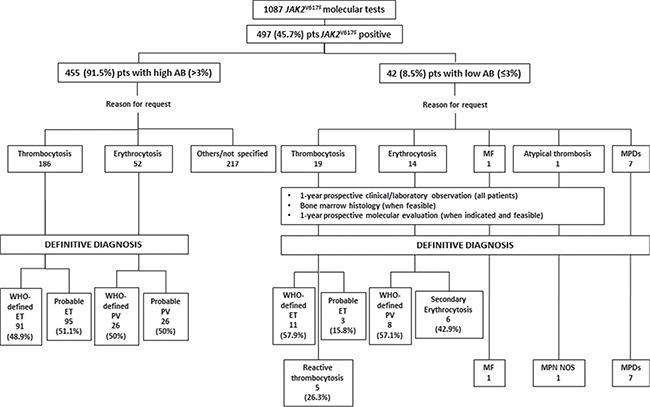
Schematic representation of the study population and the study plan ET: Essential Thrombocythemia; PV: Polycythemia Vera; MF: primary Myelofibrosis; MPN NOS: Myeloproliferative Neoplasm Not Otherwise Specified; MPDs: Myeloproliferative Diseases.

Figure 1 depicts the study population and the study plan. Overall, 497 (45.7%) of the 1087 subjects that were tested for the *JAK2*^V617F^ mutation resulted positive for the mutation.

A total of 455/497 (91.5%) patients had an AB > 3% (IC 95%: 88.75–93.71, *p* = 0.05): 52 (11.4%) were tested because of erythrocytosis, 186 (40.9%) because of thrombocytosis and 217 (47.7%) for other causes (specifically 94 suspicion of MF, 3 MPN underling atypical thrombosis and 120 others/not specified). Overall, the final diagnosis of these patients was: WHO-defined ET (91/186, 48.9%), probable ET (95/186, 51.1%), PV (26/52, 50%), probable PV (50%).

The remaining 42/497 (8.5%) patients had a low (0.1–3%, median 0.59%) AB (IC 95%; 0.62–1.15, *p* = 0.05). In most cases (30 patients, 71.4%), AB was below 1%, while only 8 (28.6%) patients had an AB above 2%. Overall, the final diagnosis of these patients was: WHO-defined MPDs (7 cases), WHO-defined ET/PMF-0 (11/19, 57.9%), probable ET (3/19, 15.8%), reactive thrombocytosis (5/19, 26.3%), PV (8/14, 57.1%), secondary erythrocytosis (6/14, 42.9%).

Here we present clinical and laboratory parameters of this cohort of 42 patients with an AB ≤ 3%.

### Low AB patients suspected of MPD

By bone marrow (BM) histology, 7/42 (16.7%) patients had a confirmed diagnosis of a hematological disease that not included classical MPNs, specifically: refractory cytopenia with unilineage dysplasia (RCUD, 2 cases); refractory cytopenia with multilineage dysplasia (RCMD, 1 case); refractory anemia with excess blasts-1 (RAEB-1, 1 case); MDS with isolated del(5q) (1 case); chronic myelomonocytic leukemia (CMML, 2 cases). Median AB of these patients was 0.46% (range, 0.12–2.79%). No additional mutations in *JAK2* exon 14, *CALR* and *MPL* genes were detected in these 7 patients.

### Low AB patients suspected of MPN

**Figure 2:**
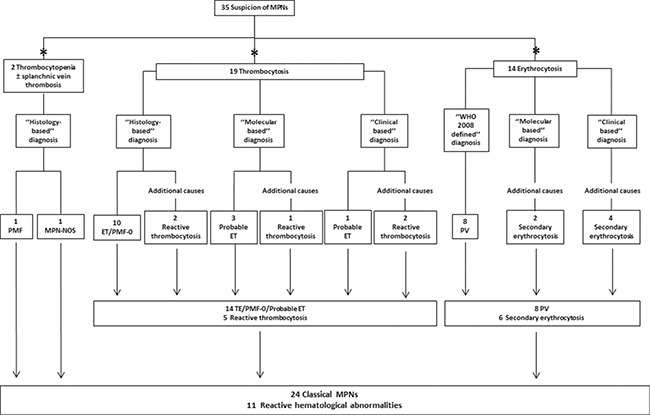
Diagnostic workflow of patients with suspected MPN and low *JAK2*^V617F^ allele burden *Main hematological abnormality motivating the *JAK2*^V617F^ evaluation. ‘’Histology-based’’ diagnosis was made when BM histology was available. In patients that were unable or refused to perform BM evaluation, prospective clinical observation and prospective molecular monitoring were crucial to direct diagnostic uncertainty, allowing to define a ‘’Clinical based’’ or ‘’Molecular based’’ diagnosis, respectively.

Diagnostic workflow of the 35/42 patients with low AB and referred to our Institutions with a suspicion of classical MPNs is shown in Figure 2. Specifically, 19 patients were evaluated for suspected ET, 14 for probable PV, 1 for probable MF and 1 for MPN underling atypical thrombosis. Consequently, they were prospectively followed over time with clinical/laboratory data. In 2/35 cases, BM biopsy confirmed the suspect of a primary MF or MPN not otherwise specified (NOS) in one subject tested after a splanchnic vein thrombosis.

**Figure 3:**
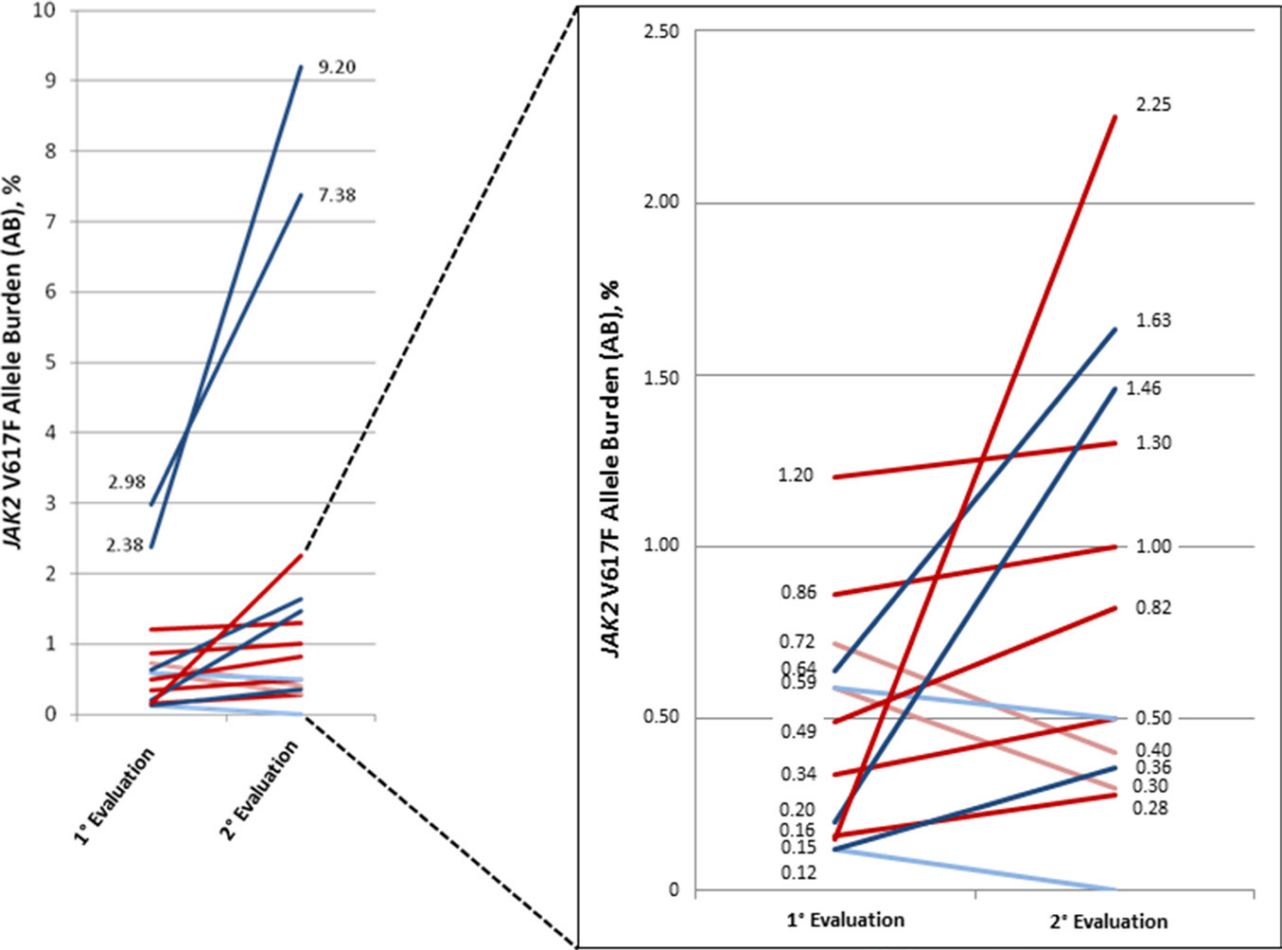
*JAK2*^V617F^ allele burden over time in patients with suspected essential thrombocytemia and polycythemia vera Fifteen patients received the second evaluation of *JAK2*^V617F^ allele burden after a period of 12 months from the first mutational test. Dark red line: final diagnosis of PV. Light red line: final diagnosis of secondary polyglobulia. Dark blue line: final diagnosis of ET. Light blue line: final diagnosis of secondary thrombocytosis.

Prospective molecular monitoring was performed in 15/35 low AB patients at a 12-month follow-up A significant increase of *JAK2*^V617F^ AB over time was observed in 11 cases (*p* < 0.05). Indeed, the median value of *JAK2*^V617F^ AB at diagnosis and during follow-up was 0.49% (range, 0.12–2.98) and 1.3% (range, 0.28–9.2), respectively. In the remaining 4 cases, a slight decrease of *JAK2*^V617F^ AB over time was registered (Figure 3).

Among the 19/35 (54.3%) subjects with low AB that received *JAK2*^V617F^ evaluation for thrombocytosis, 14 (73.7%) patients finally had a clinical diagnosis of MPN (Table 1). In 10 cases, the diagnosis was confirmed by BM histology (patients T1–T8, T10 and T12). In one case, an additional Type 1 *CALR* mutation was detected with an AB of 42% (patient T9). Conversely, three additional patients refused or were unable to perform BM biopsy, therefore were classified as ‘’Probable ET’’ because presented a persistent thrombocytosis in absence of other causes (patients T11,T13–T14). In two of these patients (T11 and T14), *JAK2*^V617F^ mutation load was reassessed after a 12-month follow-up and an increased AB was observed, corroborating the diagnosis of MPN (Table 1 and Figure 3); in the remaining case (T13), prospective molecular evaluation was not performed because cytoreductive therapy was already ongoing. Indeed, despite the absence of BM biopsy, cytoreductive therapy was administered according to standard criteria for treatment start (e.g.: age > 60 years and/or previous thrombosis and/or massive thrombocytosis). In the remaining 5 patients (patients T15–T19), thrombocytosis was transitory and was finally considered as secondary to an inflammatory disease (erysipelas, 1 case; rheumatoid arthritis, 2 cases) or to iron deficiency (2 cases). In two of these cases, BM biopsy excluded a hematological disease (patients T18 and T19). Accordingly, a decrease of AB was observed at second evaluation in patients T17 and T18.

**Table 1:** Main baseline characteristics and clinical outcome of patients investigated for thrombocytosis

Patient	Age, gender	Prior Thrombosis (Y/N)	Plt count ≥ 450 (× 10^9^/l)	BM histology	*JAK2^V617F^* AB, % 1st evaluation	*JAK2^V617F^* AB, % 2nd evaluation	CALR	*Fulfilled 2008 WHO diagnostic criteria* *(Y/N)*	Final diagnosis	Therapy	Status at last follow-up
T1	20, F	N	Y	Y	0.12	0.36	WT	Y	ET	IFN	PLT < 400
T2	46, M	Y	Y	Y	0.79	-	WT	Y	ET	IFN	PLT < 400
T3	33, F	N	Y	Y	0.20	1.46	WT	Y	ET	-	PLT < 1000
T4	25, F	N	Y	Y	2.37	-	WT	Y	ET	ASA	PLT <1000
T5	62, M	Y	Y	Y	2.98	7.38	WT	Y	ET	HU, ASA	PLT < 600
T6	17, M	N	Y	Y	3.00	-	WT	Y	ET	-	PLT <1000
T7	50, F	N	Y	Y	0.43	-	WT	Y	Early-PMF	IFN	PLT < 400
T8	31, M	N	Y	Y	2.36	-	WT	Y	Early-PMF	IFN	PLT < 400
T9	46, M	N	Y	N.A.	0.56	-	Type1 (42%)	N (absence of BM biopsy)	ET	HU	PLT < 600
T10	38, F	N	Y	Y	0.41	-	WT	Y	ET	HU	PLT < 1000
T11	57, F	N	Y	N.A.	0.64	1.63	WT	N (absence of BM biopsy)	Probable ET	ASA	PLT < 1000
T12	66, M	N	Y	Y	1.05	-	WT	Y	ET	HU, ASA	PLT < 600
T13	71, F	N	Y	N.A.	1.56	-	WT	N (absence of BM biopsy)	Probable ET	ASA	PLT < 400
T14	91, F	Y	Y	N.A.	2.38	9.20	WT	N (absence of BM biopsy)	Probable ET	HU, ASA	PLT <400
T15	72, F	Y	Y	N.A.	0.32	-	WT	N (absence of BM biopsy, evidence of reactive thrombocytosis)	Reactive (reumatoid arthritis)	-	PLT < 600
T16	81, M	N	Y	N.A.	0.34	-	WT	N (absence of BM biopsy, evidence of reactive thrombocytosis)	Reactive (reumatoid arthritis)	-	PLT < 600
T17	43, F	N	Y	N.A.	0.59	0.50	WT	N (absence of BM biopsy, evidence of reactive thrombocytosis)	Reactive (iron deficiency)	IRON THERAPY	PLT < 400
T18	25, F	N	Y	Y (normal)	0.12	WT	WT	N (normal BM histology, evidence of reactive thrombocytosis)	Reactive (erysipelas)	-	PLT < 400
T19	35, F	N	Y	Y (normal)	0.61	-	WT	N (normal BM histology, evidence of reactive thrombocytosis)	Reactive (iron deficiency)	-	PLT < 600

Patients with persistent thrombocytosis in absence of other causes that did not perform BM biopsy for histological confirmation were classified as ‘’Probable ET’’. PLT: platelet (× 10^9^/l); BM: bone marrow; N.A.: not available; ET: Essential Thrombocythemia; Early-PMF: early-primary myelofibrosis; WT: wild-type; HU: hydroxyurea; IFN: interferon-alpha; ASA: low-dose aspirin. Patient 19 had received splenectomy for a previous diagnosis of immune thrombocytopenia. Only in 5 cases, lactate dehydrogenase (LDH) was elevated. No patients had splenomegaly.

The 14 patients with a final diagnosis of MPN (including WHO-defined ET and “probable ET”) showed higher hemoglobin level (median (range): 13.6 (10.9–16.2) *vs* 10.3 (7.2–12.1) g/dl) and lower leukocyte count (median (range): 7.4 (4.2–13.3) *vs* 11.3 (8.2–21) × 10^9^/l) compared to the 5 patients with reactive thrombocytosis (*p* < 0.001 and *p* = 0.05, respectively). Conversely, platelet count was similar in the two groups (median (range): 650 (161–1000) *vs* 587 (490–701) × 10^9^/l; *p* = N.S.) (data not shown). The mutation load was significantly higher in MPN patients than in subjects finally diagnosed with reactive thrombocytosis (median (range): 1.18 (0.15–2.98) *vs* 0.34 (0.12–0.61) %; *p* = 0.02). Overall, no patient with reactive thrombocytosis showed an AB ≥ 0.64% (Table 1).

Among the 14/35 (40.0%) patients with low AB that performed mutational analysis due to erythrocytosis, 8 (57.1%) had a final diagnosis of MPN (Table 2). PV diagnosis was sustained by absence of other causes of polyglobulia, *JAK2*^V617F^ positivity and reduced baseline erythropoietin (EPO) levels (patients E1-E8). In patients E1-E3 and E6-E8, the diagnosis was also confirmed by the detection of an increased *JAK2*^V617F^ AB at a 12-month molecular follow-up. In the remaining 6 cases, the hematological abnormality was finally classified as secondary erythrocytosis (patients E9-E14) due to kidney cancer with increased endogenous EPO levels (patient E9) and chronic obstructive pulmonary disease (COPD, 5 patients: E10-E14). In two of these patients, the diagnosis was supported by a decrease in the *JAK2*^V617F^ AB over time (patients E13 and E14) (Table 2 and Figure 3). In the majority of these patients with diagnosis of secondary erythrocytosis, phlebotomy and/or aspirin were administered after consideration of the causes of polyglobulia and the overall thrombotic risk of the patients [40–42]. No additional mutations were found in none of the patients investigated for erythrocytosis.

**Table 2:** Main baseline characteristics and clinical outcome of patients investigated for erythrocytosis

Patient	Age, gender	Prior Thrombosis (Y/N)	Hb ≥ 18.5 g/dl (males) or Hb ≥ 16.5 g/dl (females) (Y/N)	*JAK2*^V617F^ AB, % 1st evaluation	*JAK2*^V617F^ AB, % 2nd evaluation	Reduced EPO levels	*Fulfilled 2008 WHO diagnostic criteria (Y/N)*	Final diagnosis	Therapy	Status at last follow-up
E1	54, M	N	Y	0.15	2.25	Y	Y	PV	ASA, PHLEBOTOMY	Hct control < 45%
E2	63, M	N	Y	0.16	0.28	Y	Y	PV	ASA, PHLEBOTOMY	Hct control < 45%
E3	58, M	Y	Y	0.34	0.50	Y	Y	PV	NONE	Hct control < 45%
E4	50, M	Y	Y	0.16	-	Y	Y	PV	HU, ASA, PHLEBOTOMY	Hct control < 45%
E5	70, M	N	Y	2.23	-	Y	Y	PV	HU, ASA, PHLEBOTOMY	Hct control < 45%, PLT< 600
E6	81, M	Y	N	1.20	1.30	Y	No (Hb ≤ 18.5)	PV	HU, ASA, PHLEBOTOMY	Hct control < 45%
E7	65, M	N	N	0.86	1.00	Y	No (Hb ≤ 18.5 & EPO levels not available)	PV	ASA, PHLEBOTOMY	Hct control < 45%
E8	41, M	Y	N	0.49	0.82	Y	No (Hb ≤ 18.5 & EPO levels not available)	PV	ASA, PHLEBOTOMY	Hct control < 45%
E9	59, M	N	Y	0.12	-	N	No (increased EPO levels)	Secondary (kidney carcinoma)	ASA, PHLEBOTOMY	Deceased (kidney carcinoma)
E10	67, M	Y	N	0.16	-	N	No (Hb ≤ 18.5 & normal EPO levels)	Secondary (COPD)	ASA, PHLEBOTOMY	Hct control < 48%
E11	54, M	Y	N	0.39	-	N.A.	No (Hb ≤ 18.5 & EPO levels not available)	Secondary (COPD)	PHLEBOTOMY	Hct control < 48%
E12	82, F	N	N	0.74	-	N.A.	No (Hb ≤ 18.5 & EPO levels not available)	Secondary (COPD)	NONE	Hct control < 48%
E13	77, M	Y	N	0.59	0.30	N	No (Hb ≤ 18.5 & normal EPO levels)	Secondary (COPD)	ASA, PHLEBOTOMY	Hct control < 48%
E14	72, M	N	N	0.72	0.40	N	No (Hb ≤ 18.5 & normal EPO levels)	Secondary (COPD)	ASA, PHLEBOTOMY	Hct control < 48%

Hb: hemoglobin (g/dl); Hct: hematocrit; N.A.: not available; HU: hydroxyurea; ASA: low-dose aspirin; COPD: chronic obstructive pulmonary disease.

The 8 patients with a diagnosis of PV showed higher hemoglobin level compared to the 6 patients with secondary polyglobulia (median (range): 18.8 (17.4–20.1) *vs* 16.9 (16.2–19.7) g/dl; *p* = 0.04), but presented comparable leucocyte (median (range): 7.3 (5.4–0.9) *vs* 6.2 (4.5–9.4) × 10^9^/l; *p* = N.S.) and platelet counts (median (range): 250 (186–703) *vs* 235 (166–283) × 10^9^/l; *p* = N.S.) (data not shown). Median *JAK2*^V617F^ AB was also similar in the two groups (median (range): 0.42 (0.16–2.25) *vs* 0.49 (0.12–0.74)%; *p* = N.S.). Nonetheless, 75% of PV patients carried a mutation load above the median value of 0.44% (*vs* 16.7% in patients with secondary erythrocytosis, *p* = 0.03) (Table 2).

As expected, low AB patients investigated for a suspected PV showed significantly higher median hemoglobin levels (median (range): 18.2 (16.2–20.1) *vs* 12.9 (7.2–16.2) g/dl; *p* < 0.001) and lower median platelet count (median (range): 238 (166–703) *vs* 638 (161–1000) × 10^9^/l; *p* < 0.001) compared to patients with suspected ET (data not shown). Conversely, median *JAK2*^V617F^ burden was similar in the two cohorts both considering all evaluated patients (median (range): 0.44 (0.12–.25) *vs* 0.64 (0.12–2.98) %; *p* = N.S.) and only patients with final diagnosis of PV and ET/early-PMF/MPN NOS/probable ET (median (range): 1.18 (0.16–2.25) *vs* 0.42 (0.15–2.98) %; *p* = N.S.) (Tables 1 and 2).

Overall, in 11 out of 35 (31.4%) patients the detection of a low *JAK2*^V617F^ AB was considered insufficient to make a diagnosis of MPN (Figure 2). In 6 cases, the exclusion of MPN relied only on clinical monitoring over time, with the observation that the hematological abnormalities were transient and dependent on other contributing factors. In 2 cases BM histology revealed no signs of MPNs (patients T18 and T19), and in 3 additional patients the *JAK2*^V617F^ mutation load spontaneously decreased over time (patients T17, E13 and E14). The probability to have a final hematological diagnosis was higher in patients tested for thrombocytosis who received a diagnosis of ET/early-PMF in 73.7% of the cases (*vs* 57.1% of patients with polyglobulia finally diagnosed with PV). In the former cohort, BM biopsy was fundamental for diagnosis in 62.9% of the cases; also, the detection of a concomitant *CALR* mutation was decisive in confirming ET diagnosis in one patient. Conversely, in patients tested for polyglobulia BM biopsy was never performed, thus limiting the diagnostic accuracy. However, in 15 cases the repetition of the *JAK2*^V617F^ mutation load over time was of remarkable help in the diagnostic process. Nonetheless, in the low AB cohort, the probability to carry a hematological disease directly correlated with a higher mutation load, since all patients with a *JAK2*^V617F^ AB > 0.8% were finally diagnosed with a MPN. In order to exclude under-estimation of the *JAK2*^V617F^ AB caused by hampering correct primer or probe annealing, additional mutations in *JAK2* exon 14 were investigated; however, they were not detected in any patient.

Finally, regarding clinical outcome, the frequency of thrombosis was not significantly different according to *JAK2*^V617F^ AB, in both ET and PV. In addition, when we analyzed the distribution of low and high AB patients according to risk categories, we did not find any significant correlation between the two parameters (data not shown).

## DISCUSSION

The present study investigated the role of a low *JAK2*^V617F^ AB in a cohort of subjects that received the molecular test in the suspect of MPN. All mutational studies were performed according to international recommendations over a 1-year period and patients were homogeneously followed at a single Institution. BM histology was always decisive to direct diagnosis. When histology was unavailable, molecular monitoring together with clinical observation were utmost importance.

Among the 42 patients with low (0.1–3%) *JAK2*^V617F^ AB, 7 had a confirmed diagnosis of non-classical MPNs by BM histology, 11 were classified as reactive hematological abnormalities due to the presence of additional causes of thrombocytosis (5 cases) or polyglobulia (6 cases) whereas 24 received a diagnosis of classical MPNs. *JAK2*^V617F^ mutation represents a non-driver and subclonal event in non-classical MPNs, occurring most frequently with low mutation burden [11]. As a consequence, the percentage of subjects with an AB ≤ 3 that were finally diagnosed as MPDs was higher (7/23, 30.4%) compared to MPNs 24/716 (3.3%).

Also, the present study demonstrates that within low AB patients a higher mutation load is associated with a higher probability to receive a hematological diagnosis, with an AB ≥ 0.8% always corresponding to an overt MPN phenotype. Moreover, while higher hemoglobin levels significantly correlated with a diagnosis of WHO-defined PV, platelet count was similar in patients with or without a final diagnosis of ET/early-PMF, and was therefore not indicative *per se* of a hematological disorder. Another interesting observation is that histology, when performed, was diagnostic for a full-blown disease regardless of the *JAK2*^V617F^ AB. This not only further confirms the central role of histology in MPN diagnosis, distinguishing true MPNs by small foci of clonal hematopoiesis that are not of clinical significance, but also indicate that the morphologic pattern is not strictly driven by the molecular aberrancy. As a result, evaluation of BM histology could be useful especially for patients with a suspicion of PV and an AB below 0.8% to confirm MPNs diagnosis. Accordingly, revised WHO classifications for myeloproliferative neoplasm indicated BM morphology as one of three major diagnostic criteria, also in PV [43]. Nonetheless, BM biopsy is not feasible in all patients, because of older age and/or patients’ refusal; also, the material may result inadequate for a correct histology evaluation. In these cases, the repetition of the molecular test over time timely detected the spontaneous expansion (11 cases) or reduction (4 cases) of the *JAK2*^V617F^-positive clones and significantly oriented the diagnostic process. Finally, the co-existence of additional *CALR/MPL* or *JAK2* exon 14 and exon 12 mutations was routinely excluded in all cases of thrombocytosis or polyglobulia, respectively, that present with a low *JAK2*^V617F^ AB [44]. Accordingly, many recent studies have demonstrated that *CALR* and *MPL* mutations may co-exist with *JAK2* mutations in chronic MPNs/MDS [45,46].

From the biological point of view, our clinical results seem to sustain the hypothesis that a low *JAK2*^V617F^ AB reflects the presence of a small mutated clone within an overall polyclonal hematopoiesis. Indeed, it was recently demonstrated that patients with early stage hematological malignancy may harbor distinct clones, and that such clones may arise independently [47]. Alternatively, we can also draw the assumption that, depending on whether the oncogenic hit marks the stem or the progenitor's compartments, the mutation load might be more or less enlarged. In any case, this mutational event represents an early molecular onset and is probably not sufficient *per se* to induce the malignant MPN phenotype. Consequently, the detection of low AB in one single occasion is likely not appropriate to determine the diagnosis. Therefore, the molecular monitoring over time may allow knowing whether this is a temporary clone (a condition which is likely to occur in healthy subjects) or may expand and give origin to the disease.

In conclusion, our results highlight that the detection of a *JAK2*^V617F^ mutation at low levels is difficult to be interpreted in everyday clinical practice, since not all positive patients received a hematological diagnosis. However, all patients with an AB ≥ 0.8% finally received a diagnosis of MPN; therefore, a mutation load above this cut-off may be considered very indicative for the presence of a myeloproliferative disease. Additionally, the study identified the prospective evaluation of *JAK2*^V617F^ mutation load as a convenient and non-invasive method to evaluate patients with small mutated clones in order to timely detect the expansion of clonal hematopoiesis and diagnose a full blown disease. The study should require validation in larger cohorts of patients prospectively examined with standardized molecular methods.

## MATERIALS AND METHODS

### Study population

Between January 2013 and January 2015, 1087 *JAK2*^V617F^ mutational studies were performed at the Institute of Hematology "L. e A. Seràgnoli", Bologna. The clinical suspicions that motivated the request for *JAK2*^V617F^ evaluation were: essential thrombocytemia (ET) (299 cases, 27.5%); polycythemia vera (PV) (272 cases, 25.1%); myelodysplasia (MDS) (23 cases, 2.1%); atypical thrombosis (12 cases, 1.1%); myelofibrosis (MF) (133 cases, 12.2%); others/not specified (348 cases, 32.0%) (Figure 1). Clinical and laboratory data of patients with a suspected ET, PV or MF and an allele burden ≤ 3% were prospectively monitored for 1 year. All patients provided an informed written consent in accordance with the Declaration of Helsinki for the use of remnant DNA for investigational purposes. The study was approved by the local Ethics Committee.

### Patient samples

Polymorphonuclear cells were isolated from peripheral blood samples by density centrifugation with Polymorphoprep (Axis-Shield, Scotland) [48–50]. Genomic DNA was extracted using the QIAamp DNA Blood Mini kit (QIAGEN-Werfen) on QIAcube (QIAGEN GmbH, Hilden, Germany) and was quantified with the NanoDrop spectrophotometer (Wilmington, DE, USA).

### Molecular evaluations

Molecular analyses were assessed at diagnosis or before treatment start on DNA obtained from granulocytes. When clinical, laboratory and/or histological data were not decisive for the diagnosis of MPN, mutational status was prospectively evaluated at a 12-month interval. A second *JAK2*^V617F^ molecular test was not performed in patients that had already started cytoreductive therapy due to high thrombotic risk. *JAK2*^V617F^ mutation was evaluated with ipsogen *JAK2* MutaQuant Kit, which is based on allele specific real time quantitative polymerase chain reaction (qPCR) technology on 7900 HT Fast Real Time PCR System (Applied Biosystem) [21]. The percentage of mutant *JAK2*^V617F^ allele was expressed as the ratio of *JAK2*^V617F^ copies to total copy number (CN) of *JAK2* (CN of *JAK2*^V617F^ + CN of *JAK2* wild type). Even if the lower detection limit (LOD) of the assay was 0.01% (See Supplemental File), we identified the 0.1% as cut-off of positivity as also suggested by several studies [23–24, 27–29]. All samples were tested in duplicate with both qPCR and also with digital PCR (ddPCR) to confirm the evaluation [51–52] (See Supplemental File). In addition to *JAK2*^V617F^ mutation, *CALR* and *MPL* mutations were screened for all patients to obtain a comprehensive molecular characterization and to exclude the co-existence of additional mutations [44–46, 53–54]. *CALR* exon 9 sequencing was performed by Next Generation Sequencing (NGS) approach with GS Junior (Roche 454 platform); analysis was carried out with AVA software (GRCh38 as references). *CALR* mutations identified by NGS were confirmed by Sanger sequencing. *MPL* mutations were investigated by ipsogen MPLW515K/L MutaScreen Kit and by Sanger sequencing (for MPLS505N and other secondary exon 10 mutations), as previously described [55]. Additional masking mutations in *JAK2* exon 14 were investigated by Sanger sequencing [39]. In case of clinical suspicion of PV, *JAK2* exon 12 mutations were also tested by Denaturing High Pressure Liquid Chromatography (DHPLC) and confirmed by Sanger sequencing [55–58]. Diagnoses of all hematological diseases were made according to the WHO2008 criteria [1].

### Statistical methods

Numerical variables have been summarized by their median and range, and categorical variables by count and relative frequency (%) of each category. Comparisons of quantitative variables between groups of patients were carried out by the nonparametric Wilcoxon rank-sum test. Association between categorical variables (2-way tables) was tested by the Fisher exact test or χ^2^, as appropriate. All *p values* were two-sided and statistical significance was defined as *p* < 0.05. All statistical analyses were computed with SPSS software (SPSS Inc., Chicago, IL, USA).

## SUPPLEMENTARY MATERIALS FIGURES


