# Supplementary material for: The relevance of a low JAK2V617F allele burden in clinical practice: a monocentric study
Source: Oncotarget. 2017 Mar 31;8(23):37239–49. doi: 10.18632/oncotarget.16744 (PMC5514906; doi:10.18632/oncotarget.16744)
Supplement: Supplementary file 1 [file oncotarget-08-37239-s001.pdf]

The relevance of a low *JAK2*^{V617F} allele burden in clinical practice: a monocentric study

Supplementary Materials

SUPPLEMENTARY DATA

RESULTS

Detection and quantitation of *JAK2*^{V617F} AB by both qPCR and ddPCR

Overall, 48 samples (18 at diagnosis and 15 both at diagnosis and during the follow-up) were tested for *JAK2*^{V617F} with both qPCR and ddPCR. The median mutation burden obtained was 0.59% (range: 0–9.2%) and 0.52% (range: 0–6.77%), respectively.

As the AB did not follow a normal distribution, Spearman's rho test was used for correlation analysis. As shown in Figure S1, there was a high degree of correlation between data obtained with the two methods ($R = 0.924$; $p < 0.0001$).

Limit of detection (LOD) determination

As shown in Supplementary Figure 2, both the qPCR and the ddPCR achieved a sensitivity of 0.01%

MATERIALS AND METHODS

Digital PCR

In order to demonstrate repeatability of qPCR results, all samples were tested also with digital PCR (ddPCR) assay. ddPCR was performed using the specific PrimePCR ddPCR Mutation Detection Kit Assay for JAK2 wild-type and the V617F mutation on the QX200 platform (BioRad, Milan, Italy). The data analysis was performed using the QuantaSoft analysis software and results were expressed as the fractional abundance (FA) of mutant (a) to wild type (b) template (a/a+b).

A comparison between qPCR and ddPCR was performed through a nonparametric correlation analysis (Spearman's rho) (See Supplemntary Figure 2).

Analytic sensitivity test

The Limit Of Detection (LOD) is defined as the lowest mutant concentration that can be consistently detected above the limit of blank.

In order to compare the ability of both qPCR and ddPCR of detecting diluited mutant alleles, a sensitivity curve was performed. We prepared serial dilutions (1:2, 1:10, 1:100, 1:1000, 1:5000, 1:10000) of an already quantitated *JAK2*^{V617F} mutant genomic DNA into a genomic wild-type DNA. We include also "WT only" control wells and no template control (NTC) wells. All the dilutions were tested in duplicate.

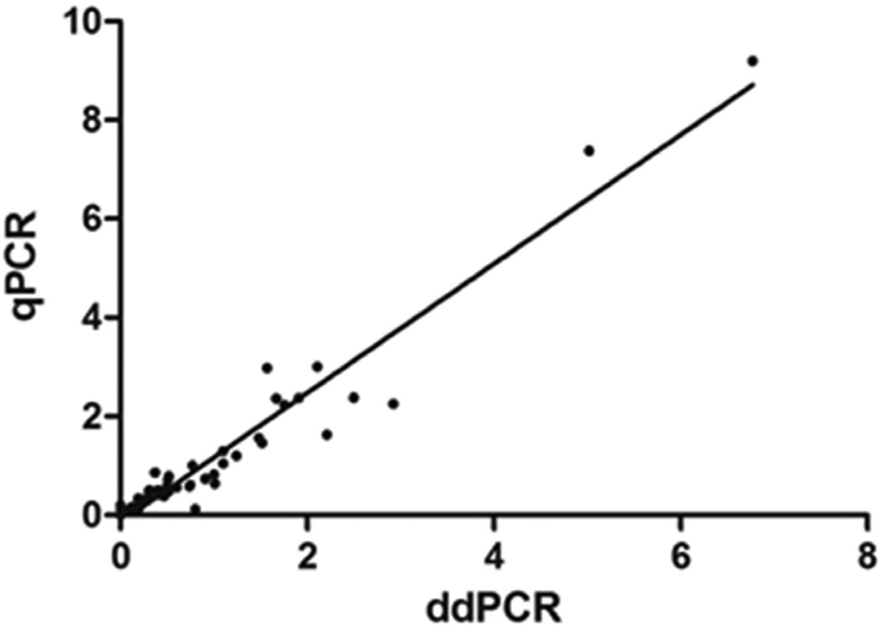

Supplementary Figure 1: Correlation between qPCR and ddPCR. Each point represents a sample where *JAK2*^{V617F} AB has been evaluated, according to qPCR (Y-axis) and dd-PCR (X-axis). The calculated Spearman's rho value is 0,924 (p < 0,0001).

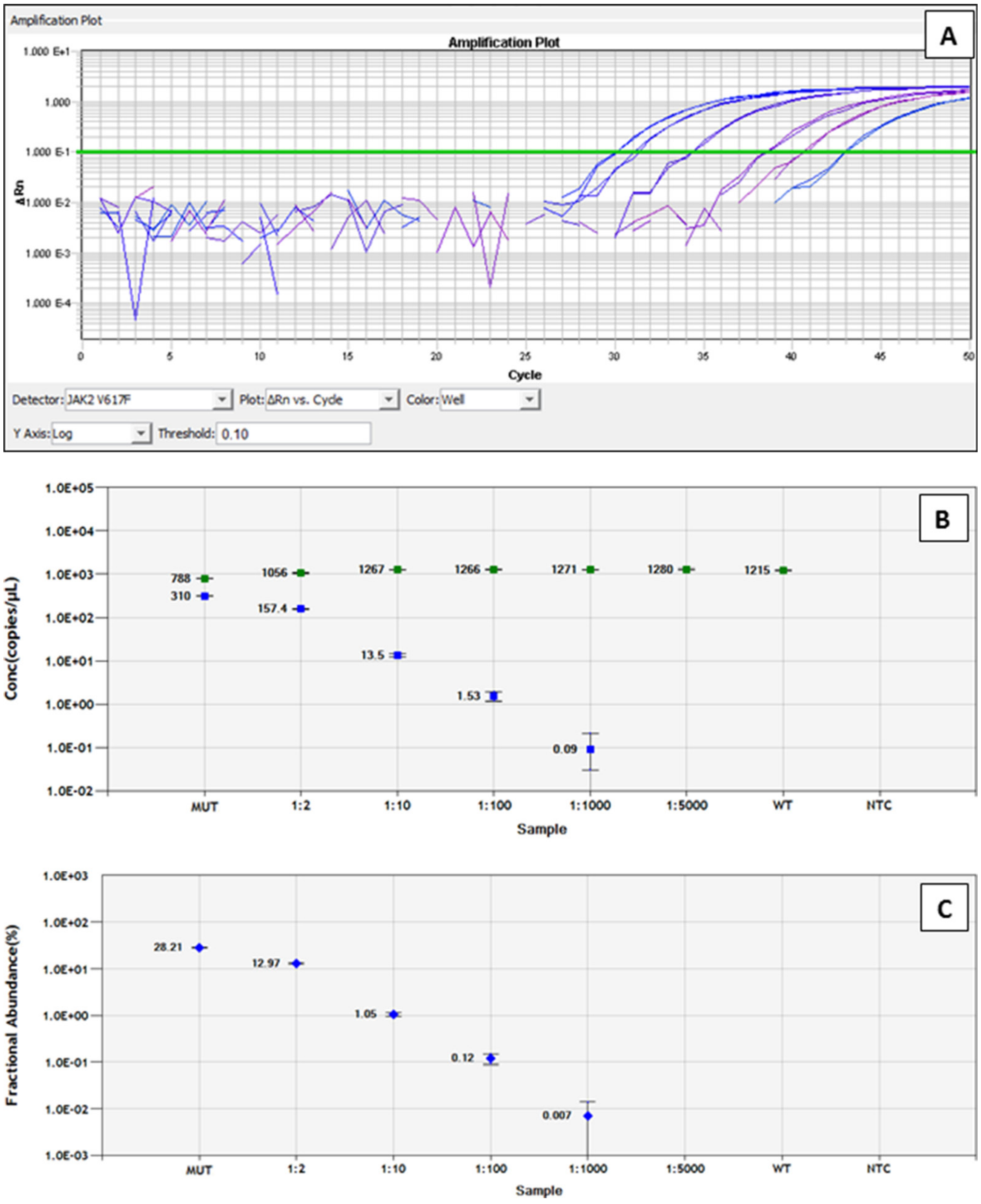

Supplementary Figure 2: Limit of detection (LOD) determination. Ipsogen JAK2 MutaQuant kit (qPCR) (**A**) and dd-PCR (**B–C**) sensitivity was assessed by quantifying a serial dilution of a mutant genomic DNA into a wild-type DNA. For simplicity, only selected dilutions of the entire standard curve (prepared as described in the Methods) are shown. (A) Amplification plot of mutant genomic DNA serial dilutions (Mut, 1:2, 1:10, 1:100, 1:1000, and WT, respectively). (B) The blue dots indicate the number of mutated copies/µl; the green dots indicate the number of wild-type copies/µl. (C) Fractional abundance plot shows the frequency of the mutant DNA into total genomic DNA.
